# Quantitative Analysis of an Intraoperative Digitalized Esophageal Heart Sound Signal to Speculate on Perturbed Cardiovascular Function

**DOI:** 10.3390/jcm8050715

**Published:** 2019-05-20

**Authors:** Young-Jin Moon, Sung-Hoon Kim, Yong-Seok Park, Jae-Man Kim, Gyu-Sam Hwang

**Affiliations:** 1 Biosignal Analysis and Perioperative Outcome Research Laboratory, Department of Anesthesiology and Pain Medicine, Asan Medical Center, University of Ulsan College of Medicine, 88, Olympic-ro 43-gil, Songpa-gu, Seoul 05505, Korea; yjmoon@amc.seoul.kr (Y.-J.M.); parkys@amc.seoul.kr (Y.-S.P.); jaemankims@gmail.com (J.-M.K.); kshwang@amc.seoul.kr (G.-S.H.); 2 Health Innovation Bigdata Center, Asan Institute for Lifesciences, 88, Olympic-ro 43-gil, Songpa-gu, Seoul 05505, Korea

**Keywords:** hemodynamic function, phonocardiogram, cardiovascular monitoring, anesthesia

## Abstract

Although visualization of heart sounds, known as phonocardiography, provides valuable information on cardiovascular hemodynamics, its use has not been widely encouraged due to the scarcity of information on its interpretation. In the present study, using the intraoperative phonocardiogram recorded by an esophageal stethoscope, we quantitatively evaluated the time and frequency domains of modulation of the heart sounds components and their association with left ventricular contractility and systemic vascular resistance under the effects of various cardiovascular drugs. We analyzed 29 pairs of intraoperative digitalized phonocardiographic signals and their corresponding hemodynamic data before and after cardiovascular drug administration (ephedrine, esmolol, phenylephrine, and/or nicardipine) in 17 patients who underwent liver transplantation. The S1 and S2 components of the heart sounds (the first and second heart sounds, respectively) were identified and their modulation in time and frequency domains was analyzed. As an index of cardiovascular function, systolic tissue Doppler wave velocity (TDI S’), maximal dP/dt from the arterial waveform, and systemic vascular resistance were simultaneously evaluated. Ephedrine/esmolol and phenylephrine/nicardipine primarily affected the S1 and S2 components of the heart sounds, respectively. This result implies that the intraoperative phonocardiogram may have the potential to be useful in detecting the changes in contractility and afterload that commonly occur in patients receiving anesthesia. In an era of constant need for noninvasive hemodynamic assessment, phonocardiography has the potential for use as a novel and informative tool for monitoring of hemodynamic function.

## 1. Introduction

Since the invention of the stethoscope by Rene Laennec in 1816, auscultation of heart sounds has been widely used in the diagnosis of various diseases, particularly in cardiology. In the field of clinical anesthesia, the esophageal stethoscope has been used for more than 60 years to listen to heart sounds continuously during surgery [1], and has been highly recommended to help ensure patient safety. However, cardiac auscultation involves subjective judgment by the clinician, which introduces variability in the perception and interpretation of the sounds, leading to differing diagnostic accuracy. Therefore, in contemporary clinical practice, its use is comparatively reduced and it has been replaced by more advanced diagnostic devices including echocardiography.

With the recent development of signal processing technology, digitalized analysis of heart sounds became available. A graphic recording of heart sounds, phonocardiography, enables more objective and quantitative analysis and interpretation. Phonocardiography provides valuable information about the function and integrity of the heart valves and about the hemodynamics of the heart, and has great potential as an aid to the diagnosis of various heart diseases [2,3,4]. Heart sounds result from the interplay of the dynamic events associated with the contraction and relaxation of the atria and ventricles, valve movement, blood flow, and vascular (aorta) dynamics. However, studies investigating the details of the hemodynamic disturbances caused by various cardiovascular drugs using intraoperative phonocardiographic data are still limited [5,6,7]. Because various kinds of vasoactive drugs have specific mechanisms of action, we can speculate on the underlying causes of sudden hypotensive or hypertensive episodes. We therefore investigated the use of intraoperative phonocardiography associated with different vasoactive drugs (positive/negative inotropes, vasoconstrictors /vasodilators), analyzed the data obtained quantitatively, and compared them with reference data such as Swan-Ganz catheter readings (systemic vascular resistance, SVR), arterial waveform (femoral dP/dt), and the results of echocardiography (contractility).

## 2. Methods

This observational study was approved by the Institutional Review Board of Asan Medical Center (2014-0357). After obtaining informed consent from each patient, we prospectively recruited patients who underwent living-donor liver transplantation consecutively with concomitant monitoring of intraoperative transesophageal echocardiography as a routine monitoring, and analyzed the recorded signal data in a retrospective fashion. In the current study, we included patients who were given bolus doses of cardiovascular drugs such as ephedrine, esmolol, phenylephrine, or nicardipine, as a routine treatment of hemodynamic instability during liver transplantation from our database. Patients with arrhythmia, valvular heart disease, heart failure with an ejection fraction <40%, or pulmonary hypertension were excluded.

All patients received standardized anesthetic care according to the protocols of our institution, as previously described [8,9,10,11]. Anesthesia was induced with propofol, midazolam, fentanyl, and rocuronium. Continuous infusion of fentanyl and 4–5 vol% desflurane in 50% oxygen/air mixture was used for maintenance of anesthesia. Radial and femoral arterial catheters were placed for continuous arterial blood pressure (BP) monitoring. A pulmonary arterial catheter (Swan-Ganz CCOmbo CCO/SvO2/CEDV, Edwards Lifescience, Irvine, CA, USA) was inserted as a part of routine monitoring. Transesophageal echocardiography was routinely used throughout the surgery for precise hemodynamic management unless the Sengstaken–Blakemore tube was placed because of active variceal bleeding or endoscopic variceal ligation had been performed within 2 weeks before the surgery. The esophageal stethoscope was inserted and positioned at a depth of 28–32 cm from the upper incisor where both S1 and S2 heart sounds were clear [12]. Heart sound signals were acquired with a custom-built replica of a phonocardiography amplifier that was connected to the end of the esophageal stethoscope [11]. These continuous beat-to-beat signals were recorded with a 1000 Hz sampling rate in a Windaq application (DATAQ Instruments, Akron, OH, USA).

The patient’s hemodynamic state was assessed by an attending anesthesiologist, who was unaware of this study, whenever hemodynamic instability was observed. The decision to administer a bolus dose of cardiovascular drug was taken by the attending anesthesiologist based on the patient’s hemodynamic state. If the patients needed fluid replacement or continuous drug infusion, the attending anesthesiologist administered treatment as appropriate according to our institutional protocol. A skillful independent anesthesiologist assessed ventricular contractility before and after administration of the cardiovascular drugs. To assess left ventricular contractility, systolic tissue Doppler wave velocity (S’) of the lateral mitral annulus was measured using transesophageal echocardiography [13]. The maximal dP/dt was also derived from the recorded femoral arterial waveform [14,15].

**Figure 1:**
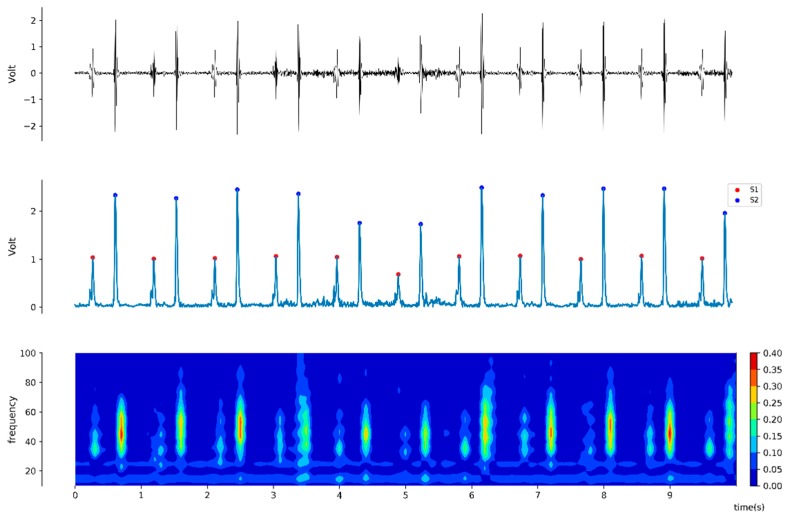
Representative plot of intraoperative phonocardiogram (upper) and consecutive signal processing for quantitative analysis. After Hilbert transformation, the S1 (red) and S2 (blue dot) components can be identified and their amplitude can be measured (middle). In spectral view, power can be visualized and calculated (lower panel).

For the offline analysis, a pair of heart sound 20-second interval windows synchronized with vital signs were collected at two minutes before and after drug administration. For each obtained 20-second window of heart sound signal, the average amplitudes of S1 and S2 heart sound were calculated by using the Hilbert transformation (Figure 1), which is derived from following equation [16]:

(1) $$H\left[x\left(t\right)\right]=\;\hat{x}\left(t\right)=\frac{1}{\pi }\int x\left(t\right)\frac{1}{t-\tau }d\tau$$

Power spectrum was also generated using fast Fourier transformation with a Hamming window. Signal processing was conducted using the CALC package of Advanced CODAS software (version 3.25, Windaq, DATAQ Instruments, Akron, OH, USA), Matlab (The MathWorks, Natick, MA, USA), Python packages, and DADiSP (DSP Development, Cambridge, MA, USA) [11,17].

### Statistical Analysis

Values are expressed as numbers (percentages), mean ± standard deviation, or median (interquartile range) according to the normality of the data. The paired t test or the signed-rank test was used for comparison of the heart sound signal and hemodynamic variables before and after drug administration. The relationship between the heart sounds and the hemodynamic variables were evaluated using Pearson’s correlation coefficient (*r*^2^). The *p*-values < 0.05 were considered significant. All statistical data analyses were performed using SPSS version 22 (SPSS Inc., Chicago, IL, USA) and R software version 3.3.2 (http://www.r-project.org).

## 3. Results

During the period of investigation, 26 patients were screened for recruitment into our study. Nine patients were excluded from the analysis: three patients with poor signal quality heart sounds, one patient with pulmonary hypertension, and five patients with incomplete data recordings. Therefore, 17 patients were included for analysis. Table 1 shows the patients’ baseline characteristics.

We identified twenty-nine fully-recorded episodes of cardiovascular drug administration from the 17 selected patients. The cardiovascular drugs used were ephedrine (*n* = 9), esmolol (*n* = 11), phenylephrine (*n* = 7), and nicardipine (*n* = 2). Duplicated patients were included only when a one hour or longer wash out period between drug administrations was guaranteed.

**Figure 2:**
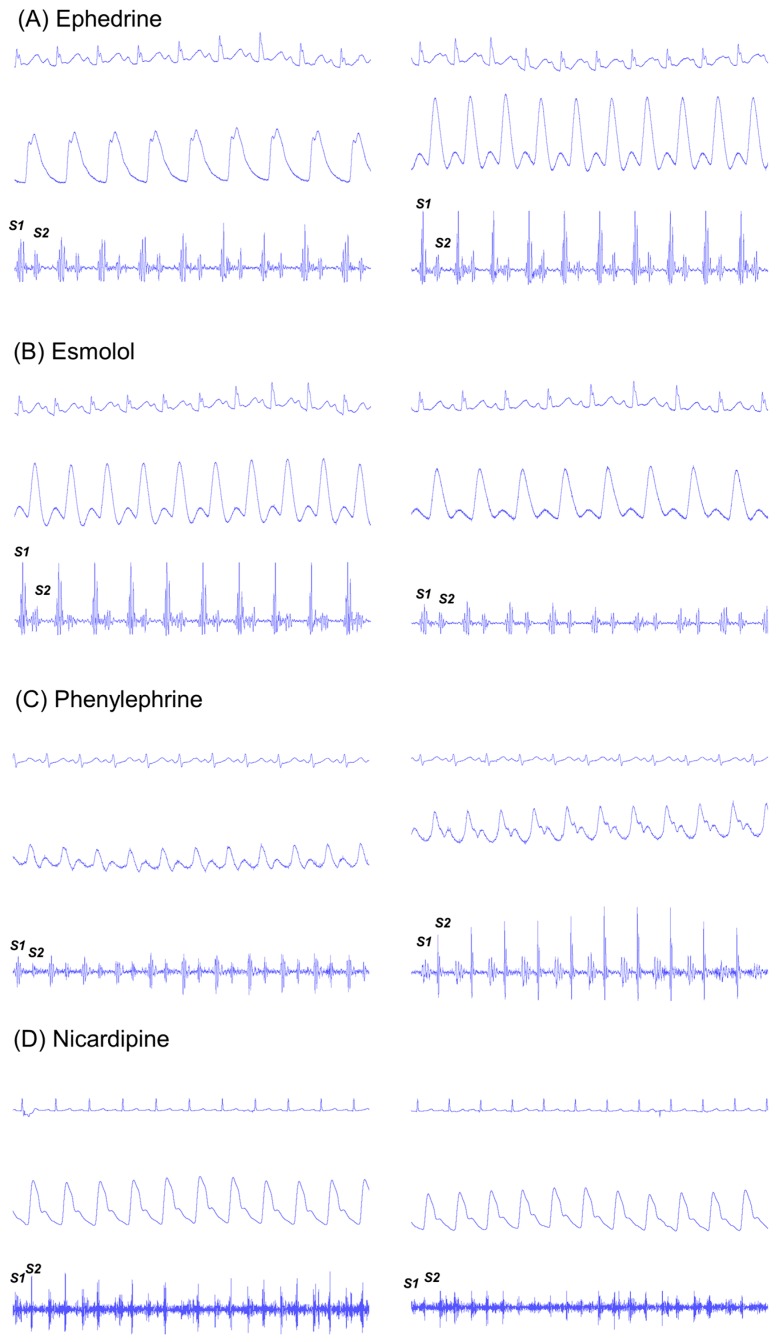
Consecutive changes in electrocardiogram (upper), arterial pressure (middle), and esophageal heart sound signals (lower panel) before (left) and after (right panel) the administration of ephedrine (**A**), phenylephrine (**B**), esmolol (**C**), and nicardipine (**D**). Administration of ephedrine increased heart rate from 56 bpm to 73 bpm, while esmolol decreased it from 69 bpm to 61 bpm. Note the changes in the S1 heart sound before and after administration of ephedrine and esmolol. Administration of phenylephrine and nicardipine changed blood pressure from 81/57 mmHg to 127/88 mmHg and from 166/86 mmHg to 105/48 mmHg, respectively. In contrast to the ephedrine and esmolol, administration of phenylephriµne and nicardipine mainly changed the S1 heart sound.

Changes in hemodynamic and phonocardiographic variables according to the type of drug administered are displayed in Figure 2 and Table 2.

**Figure 3:**
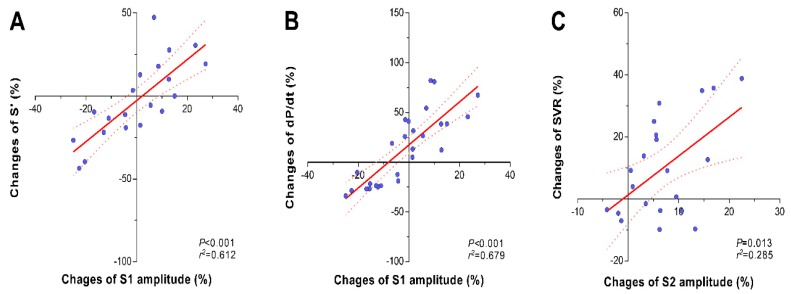
Relationship between the changes in the S1 and S2 components of heart sounds and their corresponding referential hemodynamic changes in cardiac contractility (S’ (**A**), dP/dt (**B**)), and systemic vascular resistance (**C**).

Changes in S1 heart sound were associated with changes in contractility variables. Administration of ephedrine and esmolol mainly affected the amplitude of the S1 heart sound as well as the systolic tissue Doppler wave velocity (TDI S’) and dP/dt (Table 2). Percentage changes in the amplitude of the S1 heart sound were linearly correlated with percentage changes in TDI S’ (*r*^2^ = 0.612, *p* < 0.001, Figure 3A) and dP/dt (*r*^2^ = 0.679, *p* < 0.001, Figure 3B).

In contrast, phenylephrine and nicardipine administration mainly affected the amplitude of the S2 component (Table 2). Percentage changes in the amplitude of the S2 heart sound were linearly correlated with percentage changes in systemic vascular resistance (*r*^2^ = 0.285, *p* = 0.013, Figure 3C). However, the amplitude of the S1 heart sound was not affected by administration of phenylephrine.

We had two cases of nicardipine administration, but concomitant recording of the echocardiogram was unavailable. The effects of nicardipine administration on the S2 heart sound and the hemodynamics appear to be opposite to those of phenylephrine, although the number of cases was not enough for statistical analysis. Both cases of nicardipine administration showed a decrease in S2 heart sound amplitude (−14.2% with respect to baseline) along with a fall in systolic blood pressure (−27.4% with respect to baseline) and systemic vascular resistance (−43.5% with respect to baseline).

**Figure 4:**
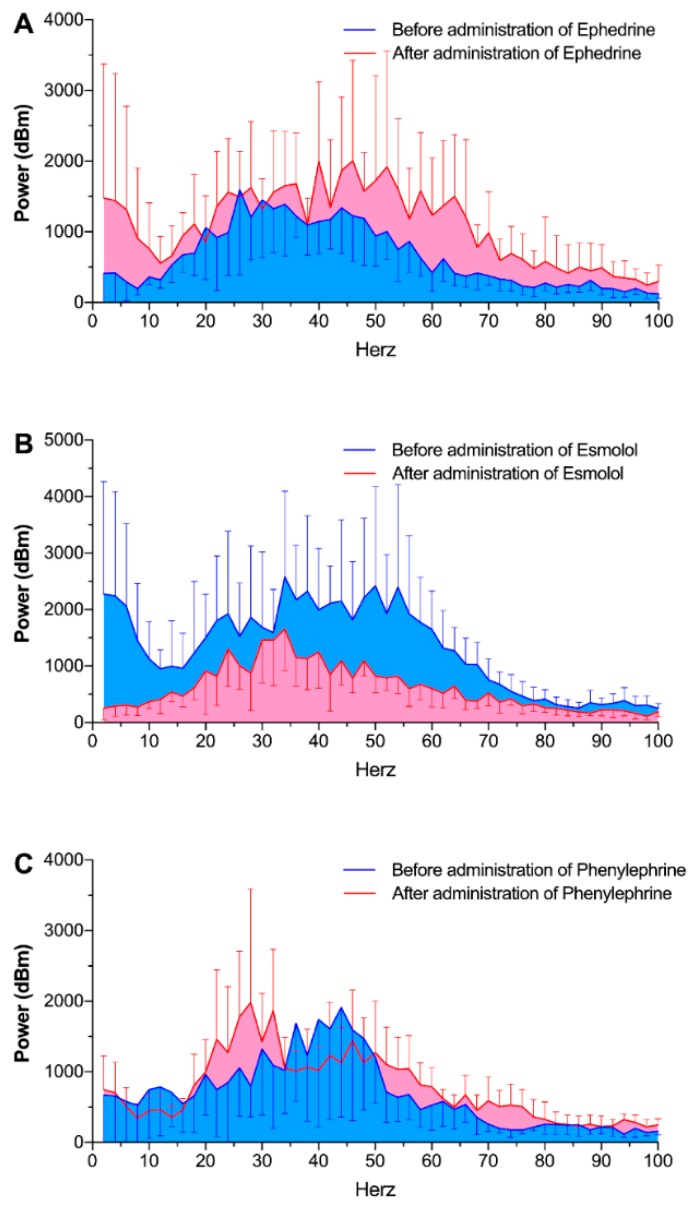
Frequency domain analysis of heart sound signals before (blue) and after (red) the administration of ephedrine (**A**), esmolol (**B**), and phenylephrine (**C**).

Power spectral analysis of the heart sounds also revealed similar results. Total power of the heart sounds significantly changed after administration of ephedrine (from 47.1 ± 2.9 dBm to 75.7 ± 5.9 dBm, *p* = 0.013) and esmolol (from 83.6 ± 5.3 dBm to 44.8 ± 2.1 dBm, *p* = 0.001, Figure 4). Phenylephrine administration did not alter the total power of the heart sounds.

## 4. Discussion

In this study, we quantitatively measured the changes in intraoperative heart sounds according to the administration of various vasoactive drugs. The administration of ephedrine/esmolol and phenylephrine/nicardipine had independent effects on the S1 and S2 heart sound components, respectively. Of interest, it appears that changes in the S1 heart sound mainly reflected cardiac contractility changes caused by positive/negative inotropes. In contrast, the S2 heart sound was mainly associated with changes in SVR, which were brought about by the vasoconstrictor/vasodilator drugs. All these changes were reaffirmed by concomitant use of echocardiography (TDI S’), systolic arterial pressure waveforms (dP/dtmax), and Swan–Ganz monitors (SVR) as standard references. Our results suggest that digitalized heart sound analysis may have the potential to discriminate between different physiological mechanisms causing hemodynamic malfunction, for example, in the differential diagnosis of hypotension due to reduced cardiac contractility or reduced SVR.

Our results suggest that cardiac contractility changes during the use of ephedrine or esmolol mainly affect the S1 heart sound, but not S2. Although we do not have the exact explanation for these findings, we can review and speculate on the underlying physiology. During the cardiac cycle, the first sound (S1) is mainly generated by the closing of the atrioventricular valves [2]. It is known that the S1 sound includes four major components: The initial vibration occurs when the first contraction of the ventricle moves blood towards the atria, closing the atrioventricular valves. The second component is caused by the sudden tension of the closed atrioventricular valves, decelerating the blood flow. The third component represents oscillation of blood between the root of the aorta and the ventricular walls, and the fourth component involves the vibrations caused by turbulence in the blood ejected into the great vessels. It is plausible that increased cardiac contractility results in increased tension being applied to the atrioventricular (AV) valves, which results in amplification of the S1 sound (this would be expected to increase the amplitude of at least the first component of S1 mentioned above, since ventricular systole is the primary force that closes the AV valves). Our results show that ephedrine augmented the S1 amplitude to +12.7% from the baseline (*p* = 0.012), while esmolol attenuated the S1 amplitude to −15.3% from the baseline (*p* < 0.001). These changes are consistently accompanied with TDI S’ and femoral dP/dtmax changes, and were linearly correlated with percentage changes of TDI S’ (*r*^2^ = 0.612, *p* < 0.001) and dP/dtmax (*r*^2^ = 0.679, *p* < 0.001). This effect can be explained by the fact that esmolol is a β1-selective adrenoceptor antagonist and, therefore, by blocking cardiac β-receptors, would be expected to have a negative inotropic effect, thus reducing the force of cardiac contraction. Ephedrine is an adrenoceptor agonist that acts on both α and β adrenergic receptors at the post-ganglionic neuron/effector junction, and also facilitates the release of norepinephrine (NE) from the synaptic vesicles of this neuron. Therefore, both its direct action on β1 receptors in the sino-atrial node (resulting in positive chronotropic and inotropic effects), and its indirect sympathomimetic effect via the release of NE would explain this augmentation of the amplitude of S1. Its α-agonist effects would be expected to cause vasoconstriction with an effect on the S2 component similar to that of phenylephrine (which, in fact, was not observed).

In this study, we used femoral dP/dtmax and TDI S’ as representative measurements of cardiac contractility. Our current data showed that the changes of dP/dt and the changes of TDI S’ also correlated. Although left ventricular (LV) dP/dtmax is used as a variable for assessment of LV contractile function, it is not feasible in the usual clinical setting. The dP/dt is not a gold standard method for measuring the LV contractility, but it is also true that dP/dt is considered to be one of the most accurate methods [15], especially when it is measured in femoral arterial pressure [18]. Therefore, we used femoral dP/dtmax as a substitute for LV dP/dtmax. It has also been reported that changes in femoral dP/dtmax accurately reflected changes in LV dP/dtmax during various interventions, though dP/dtmax underestimated LV dP/dtmax [15]. The TDI S’ is known to be a reliable echocardiographic variable that is capable of measuring even small changes in cardiac contractility [19]. However, routine use of intraoperative echocardiographic monitoring for low risk surgery is not recommended, especially if the purpose is only to measure cardiac contractility. Furthermore, TDI has an inherent limitation as a routine monitoring tool because even a skillful practitioner can only measure it intermittently. In this respect, we propose that the S1 heart sound has advantages that might make it a suitable continuous indicator for cardiac contractility that might be displayed in figures with a trend indicator.

In our study, changes in SVR seemed to be closely associated with the S2 heart sound, but not with S1. Physiologically, the second sound (S2) represents the end of systole and the beginning of diastole, and is heard at the time of closing of the aortic and pulmonary valves. S2 is probably the result of oscillations in the cardiovascular system caused by deceleration and reversal of flow into the pulmonary artery and the aorta. Therefore, it is plausible that changes in afterload would affect the amplitude of S2 rather than S1. In our study, phenylephrine and nicardipine administration significantly affected the amplitude of S2, but not that of S1. Phenylephrine significantly augmented the amplitude of the S2 heart sound (*p* = 0.016), along with systolic blood pressure (*p* = 0.014) and systemic vascular resistance (*p* = 0.007). Percentage changes of amplitude of the S2 heart sound were linearly correlated with percentage changes in systemic vascular resistance (*p* = 0.013). Interestingly, the amplitude of the S1 heart sound was not affected by administration of phenylephrine (*p* = 0.722). We had two cases of nicardipine administration; however, concomitant recording of the echocardiogram was unavailable and the number of cases was not sufficient for statistical analysis. The effects of nicardipine administration on the S2 heart sound and hemodynamics appear to be opposite to those of phenylephrine administration. We regret that we were not able to enroll more cases of nicardipine usage. However, our study protocol was principally based on routine patient care and most cases showed low SVR during liver transplant surgery. It is unethical to use nicardipine if it is not indicated clinically. Although we refrained from making any inferences from this deficient data, certain predictions as to the likely result can be made from a knowledge of the mechanism of action of nicardipine. It is a dihydropyridine calcium channel blocker (of the type that have a specificity for L-type Ca++ channels). Its effects include vasodilatation due to inhibition of the contraction of vascular smooth muscle, which might result in a reduction of amplitude of the S2 component as was observed in our two cases, and a small (less predictable) positive inotropic effect due to increased sympathetic tone as a baroreceptor reflex to the vasodilatory drop in blood pressure. The predicted effect of this on the heart sounds would be a modest increase in the amplitude of S1.

Estimation of and maintenance of an adequate level of SVR is a key requirement for hemodynamic management of surgical patients. Low SVR is known to be a frequent cause of low BP during surgery [20]. The pharmacological control of SVR in order to maintain a BP sufficient to ensure adequate organ perfusion is crucial, especially in critical care patients such as those with septic shock or chronic cirrhotic liver disease [21,22,23]. However, most of the existing commercial monitoring devices cannot directly measure the SVR. Instead, those devices are focused on the estimation of cardiac output, and the SVR is indirectly estimated by calculation using derived cardiac output and measured BP. For this reason, the accuracy of the derived magnitude of the SVR is dependent on the precision of cardiac output calculation. In view of this limitation, estimation of changes in SVR by simple tracking of the S2 heart sound amplitude in a non-invasive and continuous manner holds great potential value.

Phonocardiography has possibilities for detecting hemodynamic and pulmonary deterioration during the anesthesia. Cardiovascular depression from deep anesthesia might be found by phonocardiography, as the phonocardiographic sound may be muffled because of decreased contractility. The lung sound, which is also detected by esophageal stethoscope along with the heart sound, could be a valuable monitoring tool for the respiratory system during anesthesia [24]. Thus, monitoring the sounds from the stethoscope may have the potential to become a window for monitoring the heart and lungs during surgery. Further studies regarding the application of phonocardiography in clinical situations are warranted.

This report is the first trial to investigate and interpret the effects of various cardiovascular drugs on intraoperative heart sounds and their comparison with standard reference data. Therefore, a careful approach is required in interpreting our data, and the limitations of this novel analysis must be taken into consideration. Firstly, the amplitude the of S1 and S2 components is mainly dependent on the depth of the esophageal stethoscope, for the obvious reason that this parameter will be determined by the stethoscope’s distance from the relevant cardiac chambers/valves, where the sounds originate. For example, the amplitude is maximized when the probe of the esophageal stethoscope is located at 28–30 cm from the incisor for an average adult. However, there are reciprocal relationships between S1 and S2 amplitudes and the depth of the esophageal stethoscope. Although we tried to fix the depth of the esophageal stethoscope to the best of our ability in this study, the absolute value of S1 and S2 amplitude might have been influenced by this factor. Therefore, relative amplitude appears to be more important and easier to interpret than absolute amplitude. Secondly, we did not accurately titrate the dose of drugs based on the clinical response, and did not evenly distribute patients to each group. Furthermore, data for nicardipine was available for only two cases, and this was without echocardiographic measurement. This bias was inevitable and mainly attributed to the fact that the study protocol was based on routine clinical practice. Thirdly, although we used Hilbert transformation to detect peak points of S1 and S2 and calculated their amplitudes, this was not validated for phonocardiographic signal analysis. Although we used band-pass filters, which can significantly reduce noise, such processing can also induce signal distortion. In addition, instead of amplitude after Hilbert transformation, other sound parameters such as loudness or pitch could have been selected for comparison. Therefore, the different effects between signal filters/processing should be cautiously evaluated in any further studies. Lastly, the patient population of the present study was limited to cirrhotic patients undergoing liver transplantation. Patients with end-stage liver disease usually show altered hemodynamic characteristics such as low SVR and underlying cirrhotic cardiomyopathy [25,26]. Moreover, the heart is one of the most adversely affected organs in end-stage liver disease patients [27]. We excluded patients with preoperative arrhythmia, but it may appear during the surgery. Thus, the results of the current study may not apply to patients with normal cardiovascular function. Nonetheless, we believe that there is some merit in the novelty of the present analysis and its clinical usefulness. In this context, we are planning further large-scale studies to evaluate the full impact of phonocardiography in clinical anesthetics.

## 5. Conclusions

In conclusion, the S1 heart sound was mainly modulated by ephedrine/esmolol, and the S2 sound was mainly modified by phenylephrine/nicardipine, but not vice versa. These results suggest that intraoperative phonocardiography may have the potential to aid in the assessment of changes in contractility and afterload, respectively, in patients undergoing anesthesia. In the current era of constant need for non-invasive hemodynamic assessment, phonocardiography might prove to be a promising tool for novel and informative monitoring of hemodynamic status during major surgery.

## Figures and Tables

**Table 1:** Patients’ characteristics.

	All Subjects (*n* = 17)
**Demographics**	
Age (years)	56.6 ± 9.0
Male sex	20 (60.6%)
Weight (kg)	66.2 ± 12.0
Height (cm)	163.8 ± 8.8
Body mass index (kg/m^2^)	24.6 ± 3.9
Model for end-stage liver disease score	15.4 ± 7.6
**Underlying disease**	
Diabetes	11 (33.3%)
Hypertension	5 (15.2%)
**Causes for liver transplantation**	
Hepatitis virus-related liver cirrhosis	19 (57.6%)
Alcoholic liver cirrhosis	8 (24.2%)
Biliary liver cirrhosis	4 (12.1%)
Cryptogenic liver cirrhosis	2 (6.1%)
**Comorbidities**	
Intractable ascites	7 (21.2%)
Hydrothorax	5 (15.2%)
Hepatic encephalopathy	2 (6.1%)
Pneumonia	2 (6.1%)
Hepatorenal syndrome	5 (15.2%)
**Types of living-donor liver transplantation**	
Single-donor liver transplantation	31 (93.9%)
Dual-donor liver transplantation	2 (6.1%)
ABO-incompatible liver transplantation	7 (21.2%)

Values are expressed as mean ± standard deviation or numbers (percent).

**Table 2:** Characteristics of heart sound changes according to the different kinds of cardiovascular drugs.

	Ephedrine 10 mg (*n* = 9)			Esmolol 25 mg (*n* = 11)			Phenylephrine 100 µg (*n* = 7)		
	Before	After	*p*	Before	After	*p*	Before	After	*p*
**Vital Signs and Advanced Hemodynamic Variables**									
Blood pressure (mmHg)									
Systolic	109.6 ± 13.2	139.3 ± 14.0	<0.001	136.1 ± 16.8	124.4 ± 13.2	<0.001	93.7 ±13.2	111.5± 13.8	0.014
Diastolic	58.9 ± 12.1	74.5 ± 11.0	<0.001	71.1 ± 7.6	67.6 ± 5.3	0.053	52.8 ± 11.1	63.1 ± 16.7	0.029
Mean	75.8 ± 12.2	96.1 ± 11.2	<0.001	92.8 ± 10.3	86.5 ± 7.2	0.003	66.4 ± 10.3	79.2 ± 15.2	0.021
Heart rate (bpm)	92.9 ± 13.4	92.6 ± 13.6	0.172	99.0 ± 15.8	99.3 ± 16.1	0.397	108.3 ± 8.6	108.8 ± 8.6	0.076
SVR (dyne sec/cm^5^)	719.4 ± 199.2	892.6 ± 250.2	0.011	776.4 ± 256.1	750.8 ± 251.0	0.062	452.7 ± 110.0	524.0 ± 132.2	0.007
Percentage change (%)	30.9 (22.1; 35.3)			−3.7 (−4.6; −1.5)			13.3 (9.2; 20.6)		
TDI S’ (cm/s)	9.7 ± 2.1	11.3 ± 2.7	0.014	13.7 ± 4.4	10.2 ± 2.1	0.011	21.4 ± 12.0	19.2 ± 8.5	0.402
Percentage change (%)	18.0 (10.2; 27.8)			−20.5 (−33.2; −12.3)			−5.6 (−11.6; −1.1)		
dP/dt (mmHg/s)	725.2 ± 73.8	1065.5 ± 217.6	0.001	1051.5 ± 233.4	804.3 ± 151.1	<0.001	585.1 ± 106.1	744.8 ± 95.3	<0.001
Percentage change (%)	45.7 (38.5; 67.5)			−23.6 (−26.7; −20.3)			26.5 (22.5; 36.5)		
**Phonocardiographic Variables**									
S1 amplitude (dB)	28.1 ± 3.7	31.7 ± 4.5	0.001	33.2 ± 3.7	28.3 ± 3.3	<0.001	29.1 ± 5.3	29.0 ± 4.5	0.722
Percentage change (%)	12.7 (8.6; 15.0)			−15.3 (−18.6; −11.6)			−0.1 (−1.5; 1.6)		
S2 amplitude (dB)	24.1 ± 4.0	26.4 ± 3.8	0.004	25.5 ± 2.8	26.1 ± 2.8	0.140	21.9 ± 5.2	24.7 ± 4.4	0.016
Percentage change (%)	9.1 (5.6; 14.6)			3.5 (−1.7; 6.3)			5.5 (2.4; 11.7)		
Total power (dBm)	871.4 ± 361.6	1420.4 ± 768.0	0.007	1506.1 ± 719.5	846.6 ± 235.8	0.007	837.6 ± 501.3	1008.2 ± 370.4	0.148
Percentage change (%)	62.9 (27.1; 46.1)			−43.7 (−114.6; −42.5)			20.3 (−0.7; 37.7)		

Values are expressed as mean ± standard deviation, median [interquartile range], or numbers (percent). SVR, systolic vascular resistance; TDI S’, systolic tissue Doppler wave. Total power was calculated using fast Fourier transformation with a Hamming window from each 20 s of heart sound samples.
